# CUTIS MARMORATA TELANGIECTATICA CONGENITA WITH SKIN ULCERATIONS IN A NEW BORN

**DOI:** 10.4103/0019-5154.57618

**Published:** 2009

**Authors:** Rita Chatterjee, Subhendu Dey

**Affiliations:** *From the Department of Pediatrics, Bankura Sammilini Medical College, Bankura (WB), India.*

**Keywords:** *Cutis marmorata telangiectatica congenita*, *congenital vascular skin disorder*, *skin lesion improves*

## Abstract

Cutis marmorata telangiectatica congenita (CMTC) is a rare congenital disorder with persistent cutis marmorata, telengiectasia, and phlebectesia, which may be associated with cutaneaus atrophy and ulceration of the involved skin. We herewith report a full-term newborn female baby with CMTC at birth with ulceration over the extensor aspects of both the knee joints and right elbow joint. CMTC is a benign vascular anomaly representing dilatation of capillaries and veins of dermis and is apparent at birth. The baby had a reticulated bluish purple skin changes all over the body including the face and limb. Although it resembled physiological cutis marmorata, it was strikingly pronounced and definitely was unvarying and permanent. A variety of vascular malformation has been described along with this disorder. Etiology is not very clear and may be multifactorial, teratogens and genes are also been suggested. Prognoses in uncomplicated cases are good.

## Introduction

Cutis marmorata telangiectatica congenita (CMTC) was first described by a Dutch pediatrician Von Lohuizen in 1922 in Netherlands, and since his description, more than 100 cases have been reported. CMTC is an uncommonly reported disorder and sporadic in nature.

It is a congenital vascular anomaly characterized by persistent cutis marmorata and phlebectasia, and sometimes cutaneous atrophy and skin ulceration. A variety of associated defects have been described in up to 50% of cases.[1] It is usually a benign congenital skin lesion and is present at birth but may develop later on.

## Case Report

**Figure 1:**
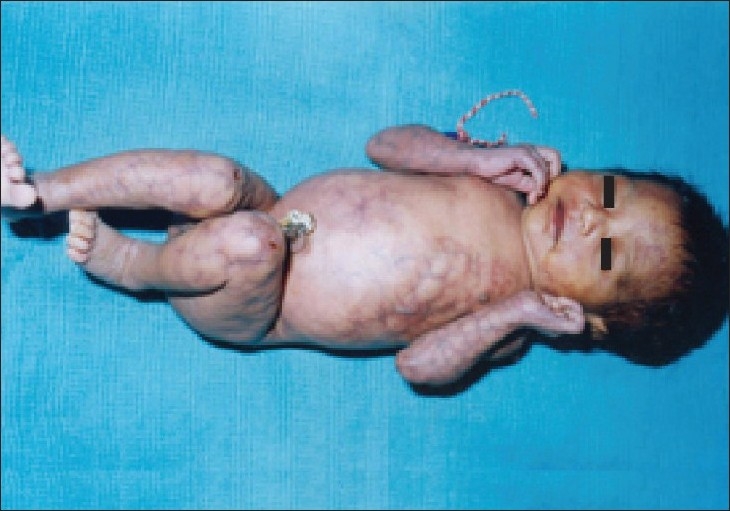
Bluish purple reticulated skin lesions involving the whole body with ulceration on knees

**Figure 2:**
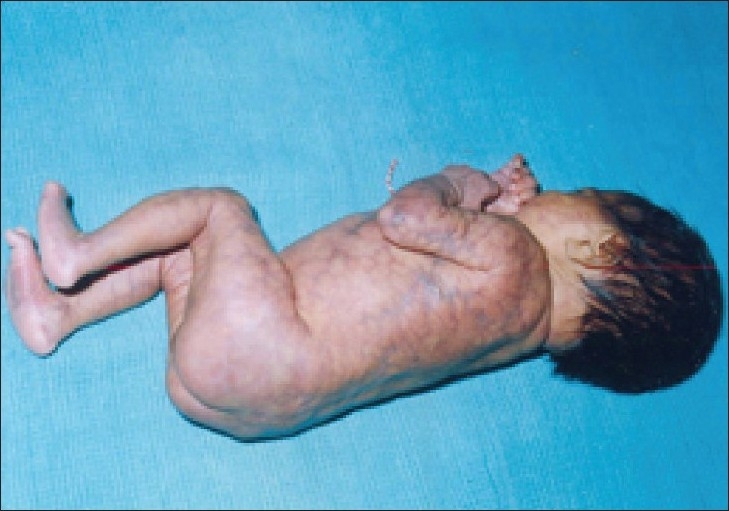
Same patient in right lateral view

**Figure 3:**
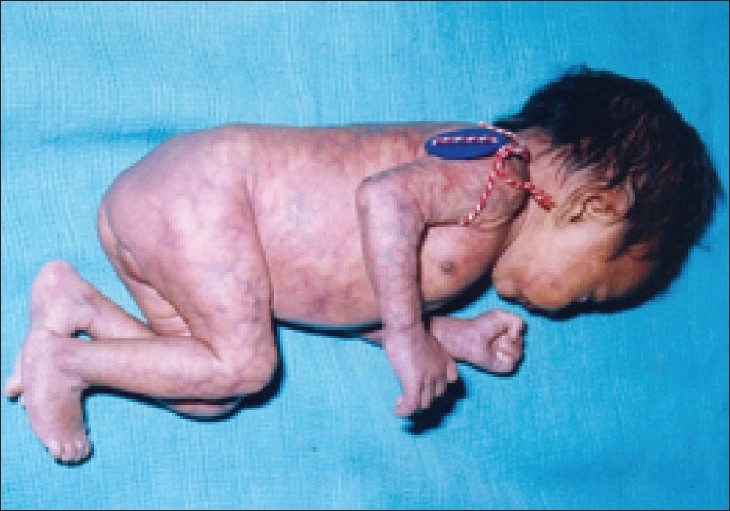
Same patient in left lateral view

A first-born female newborn delivered normally at term with uncomplicated perinatal period was found to have bluish purple reticulated skin lesions involving the whole body. The lesions were prominent over the trunk and face but were more pronounced over the limbs. In addition the baby had ulcerations over the extensor aspect of both knee joints and right elbow joint where the reticulated pattern and dilated veins were very prominent [Figures 1–3]. The involved area with reticulated marbled reddish–purple hue resembled physiological cutis marmorata but was more pronounced and was unvarying in nature. The baby had a normal facies, with head circumference of 34 cm, birth weight. 2.7 kg and there was no other vascular anomaly or asymmetry of the growth of the limbs. The systemic examination including the cardiological, neurological, opthalamological systems was normal.

## Discussion

CMTC is an infrequently reported congenital cutaneous disorder and is usually present at birth but may also appear up to 3 months to 2 years after birth. However, after its first description in 1922 by Von Lohuizen, more than 100 cases have been published worldwide.

In 1970 Petrozzi *et al.*,[2] reported the first case of CMTC in US and since then many cases associated with a variety of anomalies have been described. The frequency of this disorder is not known, it may be more common than reported because it is usually a benign disorder and most cases that are reported have associated malformations, hypoplasia[3] or hyperplasia of the limbs.

The pathogenesis of these disorders is not very clear and the cause may be multifactorial. Most cases occur sporadically although rare cases occur in families. Teratogens[4] and autosomal dominant mode of inheritance with incomplete penetrance have been considered as etiologies. Some authors suggest that lethal gene hypothesis (i.e. the lethal dominant gene survived by means of mosaisism) best explains the patchy distribution of the lesion in many cases and its sporadic occurrence. CMTC has been reported in association with fetal ascities and elevated maternal beta human chorionic gonadotropin hormone.[5]

The case reported is a female full-term newborn, which had an uncomplicated perinatal period having persistence cutis marmorata, telengeictasia, and phlebectasia with ulcerations over the extensor aspect of both knee joints and right elbow joint at birth.

Vascular anomalies, for example, Sturge–Weber syndrome,[6] Klippel–Trenaunay–Weber (KTW) syndrome have been associated with CMTC. The baby reported in this article had a normal facies with no other vascular abnormality, and systemic evaluations including opthalmological examination were normal. The baby had no asymmetry of the limbs or limb growth abnormality.

The incidence of abnormalities associated with CMTC varies from 18.8%[7]-89%.[8] The association of macrocephaly with CMTC is a subgroup of distinct disorder and may be associated with overgrowth syndrome.[9] Some cases are associated with arrhythmia and sudden death may occur, thus requiring close monitoring and this may lead to failure to thrive (FTT).[10]

Remarkable reports include atypical CMTC with retinoblastoma.[11] Interestingly, association with tumor syndromes such as meningioma or leukemia have also been reported. Uncommonly; mental retardation, aplasia cutis congenital, multicystic renal disease, glaucoma, Mongolian spot[12] and many other associations have been reported with this disorder.

A review of literature reveals controversies regarding gender-related prevalence. Several series reveal that the disorder affects more girls than boys, however the number was small and the difference was not statistically significant. A report also suggests that boys tend to have localized disease, our case was a girl child and she had generalized distribution of the lesion.

The differential diagnosis reveals several conditions such as capillary malformations,[13] KTW syndrome, neonatal lupus erythematosus, nevus anemicus, and physiological cutis marmorata, livedo reticularis associated with collagen vascular disorder, nevus flammus, and diffuse phlebectasia.

The disorder is diagnosed clinically and histopathology is not much helpful. Dilatations of capillaries and venules occur in the dermis, a proliferation of vascular channel is an atypical histopathological finding.[14] The imaging studies are indicated only for the evaluation of suspected congenital anomalies.

The disorder is self limiting and treatment is not necessary unless complicated with other associated anamolies, for example, glaucoma, multicystic kidney disease, limb asymmetry, and cardiac malformations. Consultation with orthopedists, neurosurgeons, and opthalamologists is necessary along with vascular cosmetic surgeons in some cases.

The prognosis is good with improvement of the skin lesions especially during the first two years of life and the phenomenon is attributed to maturation of the skin along with the growth of the child.
